# Assessment of Disclosure of Psychological Disability Among US Medical Students

**DOI:** 10.1001/jamanetworkopen.2020.11165

**Published:** 2020-07-23

**Authors:** Lisa M. Meeks, Melissa Plegue, Ben Case, Bonnielin K. Swenor, Srjan Sen

**Affiliations:** 1 Department of Family Medicine, The University of Michigan Medical School, Ann Arbor; 2 Wilmer Eye Institute, Johns Hopkins School of Medicine, Baltimore, Maryland; 3 Department of Epidemiology, Johns Hopkins Bloomberg School of Public Health, Baltimore, Maryland; 4 Michigan Neuroscience Institute, The University of Michigan Medical School, Ann Arbor

## Abstract

This survey study examined data from medical schools’ disability offices to estimate the proportion of students who disclose psychological disabilities.

## Introduction

According to a 2019 study,^1^ medical student disclosure of disability increased by 69% from 2016 to 2019. In a comparison of data from schools that responded both years, the largest gain was in psychological disability. To better understand whether a meaningful proportion of students with mental health diagnoses disclose their disability, we assessed the proportion of MD students reporting psychological disabilities and examined subcategories of psychological disability.

## Methods

This survey study was conducted between September 2018 and March 2019. A survey assessing the number of students reporting disabilities was sent to 140 fully accredited US allopathic medical schools’ disability offices or the school’s primary contact for disability disclosure. The study followed the American Association for Public Opinion Research (AAPOR) reporting guideline. Given the deidentified nature of the data, the University of Michigan Medical School institutional review board considered the study exempt. Aggregate data were used to estimate the proportion of medical students reporting a psychological disability and the prevalence of subcategories of psychological disability. Descriptive analyses were conducted in R version 3.5.1 (R Project for Statistical Computing). No prespecified level of statistical significance was set.

## Results

Of the 140 schools surveyed, 89 (63.6%) responded with overall categorical data for registered students with disability.^1^ Characteristics of responding schools are outlined in Table 1. Seventy-four medical schools (52.8%) provided data on primary psychological diagnosis (eg, depression, anxiety, bipolar disorder). Responding schools represented 46 635 MD students with 2189 (4.7%; 95% CI, 4.5%-4.9%) registering as having a disability. Overall, 675 MD students reported at least 1 psychological disability, which accounted for 30.8% ( 95% CI, 28.9%-32.8%) of all individuals who reported any disability and 1.4% of all students in the sample. The most frequently reported diagnoses were anxiety and depression (17.3% [379 students] and 5.8% [128 students] of all reported disabilities, respectively). MD students with a primary diagnosis of anxiety accounted for 0.8% (95% CI, 0.7%-0.9%) of the overall student population, and students with a primary diagnosis of depression accounted for 0.3% (95% CI, 0.2%-0.3%) of the total population (Table 2).

**Table 1.** Characteristics of 74 Responding Medical Schools

Characteristic	No.(%)
Public ownership	43 (58.1)
Region	
Central	23 (31.1)
Northeast	24 (32.4)
Southern	14 (18.9)
Western	13 (17.6)
Financially integrated with parent university	66 (89.2)
Community based	14 (18.9)

**Table 2.** Frequency of Students Registered With Psychological Disability, 2019 Data From 74 Allopathic Medical Schools

Characteristic	Students, No.^a^	Students with disabilites, % (95% CI) (n = 2189)^b^	Total medical students, % (95% CI) (n = 46 635)^c^
Overall psychological disability	675	30.8 (28.9-32.8)	1.4 (1.3-1.6)
Adjustment disorder	14	0.6 (0.4-1.1)	0.03 (0.02-0.05)
Anxiety disorder	379	17.3 (15.8-19.0)	0.8 (0.7-0.9)
OCD	35	1.6 (1.1-2.2)	0.08 (0.05-0.1)
PTSD	44	2.0 (1.5-2.7)	0.09 (0.07-0.1)
Bipolar disorder	36	1.6 (1.2-2.3)	0.08 (0.05-0.1)
Depressive disorder	128	5.8 (4.9-6.9)	0.3 (0.2-0.4)
Eating disorder	13	0.6 (0.3-1.0)	0.03 (0.02-0.05)
Cognitive disorder	21	1.0 (0.6-1.5)	0.05 (0.03-0.07)
Schizophrenia	3	0.1 (0.04-0.4)	0.006 (0.002-0.02)
Other	18	0.8 (0.5-1.3)	0.04 (0.02-0.06)

Abbreviations: OCD, obsessive-compulsive disorder; PTSD, posttraumatic stress disorder.

^a^ Subtypes do not add to total due to the possibility that some students are registered with multiple psychological disabilities.

^b^ Percentage based on the total number of MD students with disabilities.

^c^ Percentage based on the total number of MD students.

## Discussion

Research indicates that 30.8% of reported disabilities among MD students are psychological,^1^ representing an 11.9% increase in psychological disabilities during the past 3 years. While recent meta-analyses suggest that 25% to 30% of all MD students meet the criteria for depression,^2,3^ our survey estimates that only 0.3% of students disclose depression as a disability. Therefore, the proportion of disclosures is considerably lower than the expected prevalence of psychological disability among MD students, indicating a gap in mental health support–seeking among students.

Multiple factors may be driving the lower percentage of students disclosing psychological disabilities, including: (1) lack of engagement between MD students and mental health services, reducing opportunities for referral; (2) lack of stakeholder awareness that mental health diagnosis may be eligible for accommodation under the Americans with Disabilities Act^4^; (3) lack of accommodation of psychological disability in favor of placing students on a leave of absence; (4) stigma surrounding psychological disabilities^4,5^; (5) timing of diagnosis (ie, before or during medical school); (6) access to and cost of an evaluation; and (7) concerns regarding mandatory disclosure requirements on state board licensing.^6^

Limitations of this study include the exclusion of osteopathic schools in the reporting of disability, which affects the generalizability of our results to all medical students. Additionally, our survey only captured students who required accommodation. Not all students who screen positive for or are diagnosed with a psychological disability required accommodation.

While our data indicate a substantial percentage increase in disclosure of psychological disability, the number of students who disclosed psychological disabilities remained small. This suggests that many students who may benefit from accommodation are going without this support. Failure to identify, support, and address psychological disabilities may have wide-ranging implications for students. Therefore, a comprehensive commitment to the mental health of medical students must in part include recognizing psychological disability, removing barriers to disclosure, and ensuring appropriate support through accommodation.
