# Error in Byline

**DOI:** 10.1001/jamanetworkopen.2020.23226

**Published:** 2020-09-24

In the Research Letter titled “Assessment of Disclosure of Psychological Disability Among US Medical Students,”^1^ published July 23, 2020, there was an error in the spelling of an author’s name in the byline. The last author’s name should have appeared as Srijan Sen. This article has been corrected.^1^

## References

[1] Meeks LM, Plegue M, Case B, Swenor BK, Sen S Assessment of disclosure of psychological disability among US medical students. JAMA Netw Open. 2020;3(7):e2011165. doi:10.1001/jamanetworkopen.2020.11165 32701156 PMC7378748

